# Editorial: Novel immunological characteristics and immunotherapeutic targets in pancreatic cancer

**DOI:** 10.3389/fimmu.2024.1428740

**Published:** 2024-06-03

**Authors:** Husain Yar Khan, Yu Lou, Rachel E. Sexton-Bonacci

**Affiliations:** ^1^ Department of Oncology, Barbara Ann Karmanos Cancer Institute, Wayne State University School of Medicine, Detroit, MI, United States; ^2^ Department of Hepatobiliary and Pancreatic Surgery, the First Affiliated Hospital, Zhejiang University School of Medicine, Hangzhou, China; ^3^ Biochemistry and Molecular Biology, Michigan State University, East Lansing, MI, United States

**Keywords:** pancreatic cancer, immunology, immune system, editorial, PDAC

Pancreatic ductal adenocarcinoma (PDAC) is an aggressive and deadly disease with an overall 5-year survival rate of 13% (1). Current preclinical and clinical work is emerging to understand the interaction between the immune system, immunomodulatory processes and the tumor itself. Categorized as a ‘cold-tumor’, PDAC is seemingly non-responsive to immunotherapies, has a lack of T-cell infiltration, lack of tumor antigens and CD8+ T-cell exclusion (2). These factors make this disease difficult to treat compared to ‘hot-tumors’ which respond well to immune checkpoint (ICP) inhibitors and have better overall patient outcomes (3). The Research Topic, “*Novel immunological characteristics and immunotherapeutic targets in pancreatic cancer*” highlights emerging preclinical and clinical research that is aimed at modulating the tumor microenvironment (TME) and incorporating a strategy to rewire these ‘cold-tumors’ to allow for improved response rates to ICP inhibitors and address novel ways to target this disease.

Hartupee et al. has reviewed the TME of PDAC, highlighting its role in therapeutic resistance. The article describes the molecular and cellular composition of the TME and discusses the desmoplastic environment created by stromal support cells and immunomodulatory cells, which leads to a dense, hypoxic microenvironment that promotes tumor growth and immune evasion. PDAC is considered a ‘cold tumor’ owing to its limited immunogenicity, which leads to weak immune responses and results in challenges for immunotherapy. Therefore, it’s been suggested that targeting the TME alongside PDAC tumor cells can possibly convert an immunologically cold tumor into a ‘hot tumor’, overcoming immuno-therapeutic impediments. In this regard, CAR-T cell therapy, cancer vaccine therapy, use of bispecific T-cell engaging antibodies, antibody-drug conjugates, oncolytic virus and microbial therapies are some of the potential novel therapeutic strategies for effective PDAC treatment that have been discussed in detail by the authors. Understanding how to maximize these immune modulating strategies effectively by identifying patient populations or subgroups that may benefit from these treatments will be critical towards advancing an understanding of PDAC tumors and their intricate and complex interaction with the immune microenvironment.

The article by Henderson et al. explores the potential of using bacteriotherapy as an alternative approach for treating PDAC. It provides a historical overview of bacterial therapy beginning with Dr. William Coley’s pioneering work in the late 19th century and discusses how bacteria may help to stimulate anti-tumor immune responses. The review points out significant hurdles with existing treatments for PDAC, including resistance to chemotherapy and the reduced efficacy of immunotherapy, and proposes that using tailored bacteria or bacterial products could specifically target the tumor microenvironment and trigger immune responses, providing a prospective avenue for overcoming these treatment challenges and enhancing patient outcomes. The idea is to colonize tumors with bacteria, prompting the immune system to attack the bacterial antigens in the tumor instead of the tumor’s own antigens. This would result in IFN-γ and Th1 mediated immune responses, which would then lead to the development of acquired immunity that specifically targets the tumor. The article also recognizes the potential risks and limitations of bacterial therapies and underscores the importance of continued research and development in this emerging field.

Tay et al. details how two upregulated metabolic pathways, hypoxia and tryptophan metabolism, contribute to the immune suppressive nature of PDAC by modulating monocyte infiltration and loss of antigen presenting cells. This article demonstrates, using tumor 3D spheroid model and LC-MS/MS techniques, how ERO1a (hypoxia) and IDO1 (tryptophan metabolism) silencing impacts the tumor monocyte population. These findings are important and suggest that inhibition of ERO1a and IDO1 result in enhanced antigen presentation through monocyte differentiation into dendritic cells and enhanced secretion of pro-inflammatory factors leading to more effective immune responses within this disease. The study provides a systematic model to mimic the unsustainable hypoxic microenvironment *in vivo* and characterizes the immune response in a comprehensive way, which may be potentially feasible for high-throughput screening of drug responses in hypoxic microenvironment. Although the study was performed *in vitro*, further follow-up work discussed by the authors using *in vivo* immunocompetent KPC (Kras^G12D/+^; Trp53^R172H/+^; P48-Cre) mouse model will be anticipated.

The article by Qiu et al. highlights a phase II clinical trial in gemcitabine-refractory metastatic PDAC patients. Although gemcitabine monotherapy or combination are first-line treatment for PDAC, many patients progress and beyond these regimens there are few treatment options available that provide significant and substantial response rates (4). Furthermore, PDAC metastasizes to the liver frequently and has been correlated with poor survival (5). This phase II clinical trial investigates a novel therapeutic strategy which utilizes seemingly low-impact drugs in PDAC, individually, that when combined improve efficacy. The three compounds used within this trial are S-1, an anti-metabolite found to be somewhat effective in treating gemcitabine-refractory PDAC, sintilimab, a PD1 inhibitor, and anolitinib, an antiangiogenic compound. Alone, both antiangiogenic and ICPs have very limited efficacy for a variety of reasons including tumor resistance mechanisms to VEGF inhibitors and the highly suppressive tumor immune microenvironment. The basis of combining these three therapeutics was taken from clinical studies in other aggressive malignancies, such as in primary liver cancer, where this combination received substantial response and improved patient outcomes. The results of this phase II trial suggest this triple combination may contribute to partial response and stable disease in a subset of patients such as women and older patients (> 60 years). Further, a correlation between known genetic mutations, such as BRAF status, correlated to treatment response and no severe adverse toxicities were noted. Although this phase II trial had some described limitations, these results are encouraging and suggest a novel strategy to enhance both overall survival and improve response in gemcitabine-refractory PDAC patients that should be expanded upon in future studies.

## Author contributions

HK: Writing – original draft, Writing – review & editing. YL: Writing – review & editing. RS: Writing – original draft, Writing – review & editing.
